# A New Mode of Vaccination

**Published:** 1868-01

**Authors:** 


A New Mode of Vaccination.—It is performed by means of a silver ring, split in two, and having a small capsule in which is inclosed enough vaccine for thirty inoculations. The operator places the ring at the extremity of the left thumb, and thus deposits by direct application, the virus in a wound made by the right hand. According to the inventor, Dr. Carenzi, of Turin, it is possible to inoculate quicker and better than from arm to arm. It is now to be decided whether the vaccine thus preserved is more successful than that preserved by other means; it is not probable. *Union Medicale*, Oct. 5, 1867.

